# The sucrose signalling route controls Flavescence dorée phytoplasma load in grapevine leaves

**DOI:** 10.1093/jxb/erae381

**Published:** 2024-09-11

**Authors:** Cristina Morabito, Chiara Pagliarani, Claudio Lovisolo, Matteo Ripamonti, Domenico Bosco, Cristina Marzachì, Thomas Roitsch, Andrea Schubert

**Affiliations:** PlantStressLab, Department of Agricultural, Forestry, and Food Sciences, University of Turin, Grugliasco, Italy; Institute for Sustainable Plant Protection, CNR, Turin, Italy; PlantStressLab, Department of Agricultural, Forestry, and Food Sciences, University of Turin, Grugliasco, Italy; PlantStressLab, Department of Agricultural, Forestry, and Food Sciences, University of Turin, Grugliasco, Italy; Institute for Sustainable Plant Protection, CNR, Turin, Italy; PlantStressLab, Department of Agricultural, Forestry, and Food Sciences, University of Turin, Grugliasco, Italy; Institute for Sustainable Plant Protection, CNR, Turin, Italy; Dept of Plant and Environmental Sciences, University of Copenhagen, Copenhagen, Denmark; Global Change Research Institute of the Czech Academy of Sciences, Brno, Czech Republic; PlantStressLab, Department of Agricultural, Forestry, and Food Sciences, University of Turin, Grugliasco, Italy; MPI of Molecular Plant Physiology, Germany

**Keywords:** Defence-associated genes, phytoplasma disease, recovery, sugar metabolism, sugar signalling, trehalose-6-phosphate

## Abstract

Flavescence dorée (FD) is a phytoplasma disease transmitted by insects, causing severe damage in vineyards across Europe. Since there is no effective treatment, infected plants must be removed to prevent further spread. There is variation in susceptibility to FD among different grapevine cultivars, and some exhibit symptom remission, known as recovery, although the mechanisms behind this are unclear. Diseased plants accumulate soluble sugars, including sucrose, which influences the concentration of trehalose-6-phosphate (T6P), a signalling molecule affecting plant growth and stress responses. It is hypothesized that sucrose-mediated signalling via T6P could trigger defence mechanisms, reducing FD pathogen load and increasing plant recovery. To test this hypothesis, two grapevine genotypes with different susceptibility to FD were compared, revealing increased sucrose level and trehalose-6-phosphate synthase (TPS) activity in the more tolerant cultivar. However, FD-infected plants showed inhibited sucrose-cleaving enzymes and no activation of *TPS* expression. Attempts to enhance sucrose levels through trunk infusion and girdling promoted sucrose metabolism, T6P biosynthesis, and defence gene expression, facilitating symptom recovery. Girdling particularly enhanced T6P biosynthesis and expression of defence genes above the treatment point, reducing FD pathogen presence and promoting recovery. These findings indicate that elevated sucrose levels, possibly signalling through T6P, may limit FD pathogen spread, aiding in plant recovery.

## Teaser

Sucrose signalling-based regulation of trehalose-6-phosphate biosynthesis controls the establishment of grapevine defence responses to Flavescence dorée phytoplasma disease

## Introduction

Phytoplasmas are prokaryotic plant pathogens belonging to the class *Mollicutes*, associated with hundreds of hosts, including both wild and crop plants (Hogenhout *et al.*, 2008). These wall-less bacteria are transmitted by leafhopper, planthopper, and psyllid vectors (Bosco and Marzachì, 2016); they are phloem-restricted and their proliferation induces impaired phloematic transport due to phloem blockage by callose accumulation (Musetti *et al.*, 2013). Phytoplasmas produce effector proteins that interact with plant molecules (Hogenhout *et al.*, 2008; Sugio *et al.*, 2011). Phytoplasma infection severely affects morphology and physiology of host plants, inducing alterations in leaf, shoot, and root structure, photosynthetic limitation, stunted growth, virescence and phyllody of flowers, fruit desiccation, and severe growth and yield decline (Bertaccini, 2007).

Phytoplasmas are the causal agents of two major grapevine (*Vitis vinifera* L.) diseases, Bois Noir (BN) and Flavescence dorée (FD), which widely affect European viticulture yields in terms of quantity and quality. BN is caused by ‘*Candidatus* Phytoplasma solani’ (16SrXII-A, stolbur group), transmitted mainly by the cixiid *Hyalesthes obsoletus* Signoret. The FD disease was first detected in France in the 1960s and quickly spread to the most important European viticultural areas (EFSA Panel on Plant Health, 2014). Its causal agent is the FD phytoplasma (FDp) belonging to the elm-yellows group, mainly transmitted by the leafhopper *Scaphoideus titanus* Ball (Chuche and Thiéry, 2014). FDp infection causes a wide range of physiological, metabolic, and transcriptomic alterations, involving down-regulation of photosynthesis (Vitali *et al.*, 2013) and activation of potential defence responses (Margaria *et al.*, 2014; Pagliarani *et al.*, 2020; Teixeira *et al.*, 2020). However, due to the complexity of this insect–plant phytoplasma biosystem, and to the difficulty in genetically manipulating the latter two, the molecular interactions involved are still poorly characterized. FD symptoms, such as leaf downward rolling, yellowing or reddening, floral abortion and lack of cane lignification, worsen along the vegetative season and cause yield reduction and even plant death. FDp is a potential quarantine pathogen in Europe, and currently only indirect approaches are available to limit its spread, such as use of certified phytoplasma-free propagation material, removal of infected plants from the vineyard, and suppression of insect vectors (EFSA Panel on Plant Health, 2014). In the compelling search for tolerance mechanisms to FDp infection, there is an effort to develop bio-based and sustainable disease containment solutions.

All *Vitis* species investigated up to now, including *V. vinifera*, are susceptible to FDp infection, albeit showing different degrees of tolerance in the field (Kuzmanović *et al.*, 2008; Roggia *et al.*, 2014) and in controlled conditions (Eveillard *et al.*, 2016; Ripamonti *et al.*, 2021, 2022). However, no molecular markers of such differential lower susceptibility have been proposed up to now. Another aspect of grapevine tolerance to phytoplasma diseases is recovery, whereby infected and symptomatic plants undergo a natural symptom remission and a decrease of phytoplasma load. Recovery has been well documented in grapevine for both BN and FD (Morone *et al.*, 2007; Ripamonti *et al.*, 2020). The occurrence of recovery from FD in grapevine is environment- and cultivar-dependent (Morone *et al*, 2007). Interestingly, lower susceptibility to infection of healthy plants and frequency of recovery seem to follow an inverse pattern: Morone *et al.* (2007) reported that a highly susceptible genotype is more prone to recover from infection than a less susceptible one. This apparent contradiction can be resolved if molecular patterns, which are constitutively more abundant in tolerant genotypes, are elicited under severe FDp infection in susceptible genotypes. There could be possible candidates for such a role among primary (Prezelj *et al.*, 2016) and secondary metabolites (Margaria *et al*, 2014), proteins (Gambino *et al.*, 2013; Pagliarani *et al.*, 2020), and miRNAs (Chitarra *et al.*, 2018), which accumulate upon FDp infection.

Soluble sugars are straightforward candidates to control susceptibility to FD. Pathogen resistance has been associated with high sugar levels for some time (Horsfall and Dimond, 1957), and plant responses to pathogens are co-ordinately regulated with assimilate partitioning and source–sink relations (Berger *et al.*, 2004; Naseem *et al.*, 2017; Breia *et al.*, 2021), alteration of sugar concentrations and fluxes, and activation of signalling to defence responses (Roitsch and Gonzales, 2004; Proels and Hückelhoven, 2014). Soluble sugars can act as signals (Rolland *et al.*, 2006), and sucrose controls the concentration of the signalling molecule trehalose-6-phosphate (T6P; Zhang *et al.*, 2009; Figueroa and Lunn, 2016; Morabito *et al.*, 2021). Grapevines infected by BN (Hren *et al.*, 2009; Santi *et al.*, 2013) and FD (Prezelj *et al.*, 2016) phytoplasmas show altered soluble sugar status. However, no information is available on the relationship between leaf sugar concentration and either degree of susceptibility to or recovery from FD infection.

In this work, we focused on the relationship between T6P biosynthesis and expression of defence-associated genes in different systems: healthy poorly susceptible versus susceptible varieties, infected versus healthy plants, and infected plants where sucrose concentration was increased by trunk infusion treatment or girdling. We found that T6P biosynthesis was inhibited following FD infection, while it was induced in the healthy less susceptible genotype and in FD-infected plants treated to induce and increase endogenous sucrose concentration. Furthermore, girdling favoured recovery from FD. The collected data suggest that the sucrose signalling-based regulation of T6P biosynthesis controls the establishment of grapevine defence responses to FD.

## Materials and methods

### Plant material

To compare genotypes of different susceptibility to FD, 2-year-old, healthy rooted cuttings of *V. vinifera* cv Barbera (FD-susceptible) and Brachetto (FD-tolerant), both grafted onto *Vitis riparia×berlandieri* ‘Kober 5BB’ (six plant per genotype), were grown in 80 litre pots filled with a substrate composed of a sandy-loam soil–peat mixture (3:1 v/v), and randomly positioned in a vector-proof screenhouse. Each pot was fertilized once a month with a complex (20–10–10) fertilizer and irrigated twice a week to container capacity. Leaf samples were collected from each plant on 30 August 2019.

In order to investigate changes in sugar concentration and metabolism in FD-infected versus healthy plants, *V. vinifera* plants cv Barbera from three adjacent rows planted in an experimental vineyard located in Asti (44°55ʹ18.33″ N, 8°11ʹ44.05″ E) were tested for FDp infection by molecular diagnostic assays (see below) on 1 July 2018. Six randomized healthy and six FDp-positive plants were selected. Leaf samples were collected from each plant on 15 July and 15 August 2019.

Sucrose infusion treatment was performed on 12 randomized FDp-infected plants from the same vineyard. Sucrose (5% in water) was delivered by trunk infusion (Supplementary Fig. S1), on 15 and on 31 July 2019, by directly injecting the trunk xylem with a manual, drill-free instrument (Bite®, https://drp.bio/en/what-we-do/tree-care-en/bite-tree-care/). In this system, a small lenticular-shaped perforated blade entered the trunk by smoothly separating wood fibres, minimizing the perforation damage, and allowing uptake of solution by plant transpiration, avoiding cavitation caused by pressure injection. Six plants were infused with sucrose solution and six with water. Leaf samples were collected from each plant 24 and 120 h after the second treatment.

For the girdling experiment, 2-year-old, pot-grown ‘Barbera’ plants, positioned in an insect vector-proof screenhouse and cultivated as described for the genotype comparison experiment, were artificially inoculated with type FD-C FDp-infected *Scaphoideus titanus* starting on 20 June 2016. Insect rearing, FDp acquisition, and inoculation procedures were performed as previously described (Ripamonti *et al.*, 2021). The FDp-infection status on these plants was checked by molecular diagnostics assays (see below) on 10 June of the following growing season. All fruits were removed from the plants. Girdling (Supplementary Fig. S2) was then performed on 30 June 2016 on all shoots of nine randomly distributed FDp-infected plants by removing a 1 cm long bark ring midway along the plant shoot. Nine FDp-infected, non-girdled (UNG) plants served as controls. Leaf samples were collected from each girdled plant, above (PAG) and below (PBG) the girdling point, 30, 60, and 90 d after treatment. For each non-girdled plant, a single leaf sample of double size was collected randomly from the whole plant.

Each sample for FD diagnosis was composed of five randomly collected leaves, and samples for biochemical and molecular analysis were composed of four further leaves. For FD diagnosis, mid-ribs were separated from leaf blades using a scalpel. Mid-ribs or whole leaves within each sample were pooled, ground in liquid nitrogen, and stored at −80 °C.

### Flavescence dorée diagnostic assays and quantification

Total DNA was extracted from 200 mg of frozen leaf mid-ribs according to Pelletier *et al.* (2009). Molecular diagnosis was performed using a commercial kit (Detection kit Flavescence dorée and Bois Noir, Multiplex Real-time PCR system, IPADLAB), through a real-time PCR-based assay. A Taq Internal Positive Control IPC (Applied Biosystems TaqMan® Exogenous Internal Positive Control, Thermo Fisher Scientific) was added to the reaction mix, to confirm absence of contaminations potentially inhibiting the amplification process.

On the same DNA samples, relative quantification of FDp load was performed according to Roggia *et al.* (2014), and it was expressed as phytoplasma genome units per nanogram of plant DNA.

### Analysis of soluble sugar concentration

Twenty-five-milligram aliquots of frozen tissue powder from each leaf sample were extracted with 800 µl of deionized water at 70 °C for 15 min and then centrifuged at 10 000 *g* for 20 min. A 200 µl aliquot of the supernatant was diluted in 200 µl of deionized water. Glucose concentration was assessed adding 400 µl of the glucose oxidase-based reagent GAGO‐20 (Sigma‐Aldrich) to 200 µl of the diluted supernatant. The mixture was incubated at 37 °C for 30 min, then 400 µl of 12 N sulphuric acid was added to stop the reaction. Absorbance at 540 nm was measured in a 96-well flat-bottomed microtitre plate (Sartsted, Germany). The remaining 200 µl fraction of the diluted supernatant was used for determination of sucrose concentration, by incubating at 55 °C for 15 min with 15 units of invertase (I4504, Sigma-Aldrich) and determining glucose concentration as described above.

### Analysis of enzymatic activity

Protein extraction, purification, and enzymatic activity assays were performed according to Jammer *et al.* (2015) and Covington *et al.* (2016) with some modifications. One hundred and eighty milligrams of frozen tissue powder from each leaf sample was added to 25% w/w of polyvinylpolypyrrolidone and 25% w/w of Amberlite® XAD4 (Merck/Sigma-Aldrich). A total of 1.5 ml of extraction buffer (100 mM potassium phosphate, pH 7.0, 7.5 mM MgCl_2_, 20 mM MnCl_2_, 10% glycerol, 1% polyvinylpyrrolidone, 5 mM DTT, 5 mM ascorbate, 5 mM sodium bisulphite) was added to each sample, followed by shaking incubation at 4 °C for 40 min. Then, centrifugation (10 000 *g* for 15 min) allowed supernatant and pellet separation. Liquid supernatant was pipetted into dialysis tubes and dialysed twice against 20 mM potassium phosphate buffer (pH 7.4) at 4 °C. The dialysed supernatant (D-extract) was then collected and stored at −20 °C. The pellet was washed with deionized water three times and then resuspended in 1 ml of high salt buffer (40 mM Tris–HCl pH 7.6, 3 mM MgCl_2_, 15 mM EDTA, 1 M NaCl). The mixture was incubated overnight with continuous shaking; the remaining pellet was centrifuged, and the resulting supernatant was subjected to dialysis. The obtained dialysed (Z-extract) extract was collected and stored at −20 °C. Protein concentration in the extracts was determined using the Bradford method.

Activity of enzymes involved in (i) sucrose metabolism [cell wall (CWInv), cytosolic (CytInv), and vacuolar (VacInv) invertase (EC 3.2.1.26) and sucrose synthase (Susy; EC 2.4.1.13)] and (ii) hexose metabolism [hexokinase (HK; EC 2.7.1.1), fructokinase (FK; EC 2.7.1.4), phosphoglucoisomerase (PGI; EC 5.3.1.9), phosphoglucomutase (PGM; EC 5.4.2.2), UDP-glucose pyrophosphorylase (UGPase; EC 2.7.7.9), ADP-glucose pyrophosphorylase (AGPase; EC 2.7.7.27), and glucose-6-phosphate dehydrogenase (G6PDH; EC 1.1.1.49)] were assessed on supernatant or pellet extracts using spectrophotometric detection, and following the protocols further described in detail.

Specific enzymatic activity was expressed as nkat mg protein^−1^. The reaction for Susy, PGM, PGI, HK, AGPase, UGPase, and G6PDH activity was carried out at 50 °C and the increase in absorbance at 340 nm due to conversion of NAD to NADH was monitored every 30 s throughout the incubation using a plate reader (BioTek).

### Detailed protocols for assessment of enzyme activity

#### *Cell-wall*, *vacuolar, and cytosolic invertase*

Twenty microlitres of Z-extract was used to assess CWInv activity and 20 µl of D-extract was used to assess CytInv and VacInv activity in leaf, through an end-point measurement. The reaction was performed by adding 5 µl of reaction buffer pH 4.5 (454 mM Na_2_HPO_4_, 273 mM citric acid) for CytInv and VacInv and pH 6.8 (772 mM Na_2_HPO_4_, 114 mM citric acid) for CytInv, 5 µl of 0.1 M sucrose, and 20 µl of deionized water in a final reaction volume of 50 μl. Each sample was pipetted in three technical replicates in a 96-well plate (Sartsted, Germany) and one control replicate lacking substrate (sucrose). A glucose standard curve (0–50 nmol) was used to estimate enzymatic activity. The reaction mix was incubated at 37 °C for 30 min and then cooled down on ice for 5 min. An aliquot of 200 µl of GOD-POD solution [10 U ml^−1^ glucose oxidase (GOD), 0.8 U ml^−1^ peroxidase (POD), 0.8 mg ml^−1^ ABTS in 0.1 M potassium phosphate buffer, pH 7.0] was added to each well (including the standard curve). After 20 min of incubation at room temperature, absorbance was measured at 405 nm.

#### Sucrose synthase

Susy activity was measured through a two-reaction-based protocol. In the first reaction, 1 mM UDP was included in order to detect both Susy and CytInv background activity, while the second reaction, performed without 1 mM UDP, detected only the CytInv background activity. Final Susy activity was estimated by subtracting CytInv background activity from total activity, measured in the first reaction. In both reactions, 20 µl aliquots of D-extract were added to 160 µl of reaction buffer containing 1 mM EDTA, 2 mM MgCl_2_, 5 mM DTT, 250 mM sucrose, 1 mM UDP (exclusively in the first reaction mix), 1.3 mM ATP, 0.5 mM NAD, 0.672 U of HK, 0.56 U of PGI, 0.32 U of G6PDH in 50 mM HEPES–NaOH at pH 7.0. The analysis was performed in three technical replicates and sucrose was omitted in control reactions. Ninety-six-well flat-bottomed UV-Star microtitre plates (Greiner Bio One, Austria) were used for these assays.

#### Phosphoglucomutase

In order to determine PGM activity, 5 µl of D-extract from each sample was incubated with 10 mM MgCl_2_, 4 mM DTT, 0.1 mM glucose-1,6-bisphosphate (G1,6bisP), 1 mM glucose-1-phosphate (G1P), 0.25 U NADP, 0.64 U G6PDH in 20 mM Tris–HCl at pH 8.0. The analysis was performed in three technical replicates and the substrate, G1P, was omitted in control reactions. Ninety-six-well flat-bottomed UV-Star microtitre plates were used for these assays.

#### Phosphoglucoisomerase

For PGI activity measurement the reagent buffer modified from Zhou and Cheng (2008) (4 mM MgCl_2_, 4 mM DTT, 2 mM fructose-6-phosphate (F6P), 0.25 mM NAD, 0.32 mM G6PDH in 100 mM Tris–HCl at pH 8.0) was added to 5 µl of D-extract. F6P was omitted in control reactions. Ninety-six-well flat-bottomed UV-Star microtitre plates were used for these assays.

#### Hexokinase

HK activity was estimated incubating 20 µl of D-extract with 5 mM MgCl_2_, 5 mM glucose (omitted in control reactions), 2.5 mM ATP, 1 mM NAD, 0.8 U of G6PDH in 50 mM BisTris at pH 8.0. Ninety-six-well flat-bottomed UV-Star microtitre plates were used for these assays.

#### Fructokinase

Incubation of 20 µl of D-extract with a reaction buffer prepared in 50 mM BisTris at pH 8.0 (5 mM MgCl_2_, 5 mM fructose, 2.5 mM ATP, 1 mM NAD, 0.8 U of PGI, and 0.8 U of G6PDH) allowed the determination of FK activity. Fructose was omitted for control reactions. Ninety-six-well flat-bottomed UV-Star microtitre plates were used for these assays.

#### ADP-glucose pyrophosphorylase

Aliquots of 20 µl of D-extract were incubated with 0.44 mM EDTA, 5 mM MgCl_2_, 0.1% BSA, 2 mM ADP-glucose (not included in control reactions), 1.5 mM PPi, 1 mM NADP, 2 mM 3-phosphoglyceric acid (3-PG), 0.432 U of PGM, and 1.28 U of G6PDH in 100 mM Tris–HCl at pH 8.0 to evaluate AGPase activity. Ninety-six-well flat-bottomed UV-Star microtitre plates were used for these assays.

#### UDP-glucose pyrophosphorylase

In order to determine UGPase activity, 20 µl of D-extract was added to a reaction buffer in 100 mM Tris–HCl at pH 8.0 (0.44 mM EDTA, 5 mM MgCl_2_, 0.1% BSA, 2 mM UDP-glucose, 1.5 mM PPi, 1 mM NADP, 2 mM 3-PG, 0.432 U of PGM, 1.28 U of G6PDH). For control reactions, UDP-glucose was omitted. 96-well flat-bottomed UV-Star microtitre plates were used for these assays.

#### Glucose 6-phosphate dehydrogenase

For determination of G6PDH activity, 20 µl of D-extract was incubated with 5 mM MgCl_2_, 1 mM G6P, 0.4 mM NADP in 100 mM Tris–HCl at pH 7.6. G6P was omitted in control reactions. Ninety-six-well flat-bottomed UV-Star microtitre plates were used for these assays.

### Analysis of gene expression

Total RNA was extracted from 160 mg of frozen tissue powder from each leaf sample, using a cetyltrimethylammonium bromide-based protocol adapted to grapevine tissues (Carra *et al.*, 2007). RNA quantification and quality evaluation were performed using a NanoDrop 2000 spectrophotometer (Thermo Fisher Scientific). RNA integrity was further checked through electrophoresis on 1% agarose gel.

To avoid genomic DNA contamination, total RNA was treated with RNase-free DNase (Invitrogen DNase I, amplification grade, Thermo Fisher Scientific). Then, 500 ng of DNase I-treated RNA was reverse-transcribed into cDNA using the Applied Biosystems HighCapacity cDNA Reverse Transcription Kit (Thermo Fisher Scientific).

Gene-specific primers were designed with Primer3 (http://primer3.ut.ee/) (Supplementary Table S1). Targeted genes are involved in sucrose degradation and glucose-1-phosphate metabolism (*VvCWInv*, *VvSuSy2*, *VvADPase*); T6P biosynthesis [*VvTPS1A* is the most expressed trehalose-6-phosphate synthase (TPS) gene in grapevine: Morabito *et al.*, 2021], degradation [*VvT6PP* is one of the most abundant and the primarily expressed isoform in leaf among the six-member trehalose-6-phosphate phosphatase (T6PP) family in grapevine: Kerbler *et al.*, 2023], metabolic regulation, and stress responses (*VvTPS5*, *VvTPS10*); signalling (*VvbZIP11*); and defence response (*VvCAS2*, *VvSTS27*, *VvNCED1*). Annealing temperatures, % GC content, and absence of primer dimers or aspecific secondary structures were confirmed through Oligo Evaluator (Sigma-Aldrich; http://www.oligoevaluator.com/). Ubiquitin (*VvUBI*) and Actin (*VvACT1*) reference genes were used as internal controls for normalization of transcript expression levels.

RT-qPCR analysis was performed with an Applied Biosystems StepOnePlus Real-Time PCR detection system (Thermo Fisher Scientific), supported by the StepOne software, version 2.3. Reactions were carried out in a final volume of 10 µl, consisting of 1 µl diluted cDNA, 1 µl of primer mix (10 µM), 5 µl Luna Universal qPCR Master Mix (BioLabs Inc.) and 3 µl diethylpyrocarbonate-treated ultrapure water. The PCR program was set as follows: 95 °C for 10 min (initial holding stage); 45 cycles of 95 °C for 15 s, 63 °C for 1 min. Primer specificity and efficiency were assayed to ensure measurement accuracy. For melting curve analysis, the temperature was set at 95 °C for 15 s and at 63 °C for 1 min. The Δ*C*_t_ method (Livak and Schmittgen, 2001) was used to calculate normalized gene expression levels.

### Statistical analysis

Data were analysed using Student’s *t*-test or one-way ANOVA followed by Fisher’s post-hoc test when analysis of variance was significant (*P* < 0.05). Two-way ANOVA was also performed on the entire dataset to explore interaction among the considered variables. The analysis confirmed the absence of any significant interaction in most of the evaluated parameters (sugar concentration, enzymatic activity, and gene expression) in the different experiments. *P*-values obtained from the performed two-way ANOVA are listed in Supplementary Table S2, grouped by the different experiments presented in the work. SigmaPlot software (Systat Software Inc., San Jose, CA, USA) was used to carry out statistical analyses and to plot figure charts. Plants to be used as biological replicated were randomly chosen among those available; the number of replicates for each experiment is specified in the Results section.

## Results

### Comparison of genotypes with different degrees of susceptibility to Flavescence dorée phytoplasma

We contrasted healthy plants of two *V. vinifera* cultivars respectively displaying high (‘Barbera’) and low (‘Brachetto’) susceptibility to FD, as previously demonstrated in controlled conditions (Ripamonti *et al.*, 2021).

**Fig. 1.**
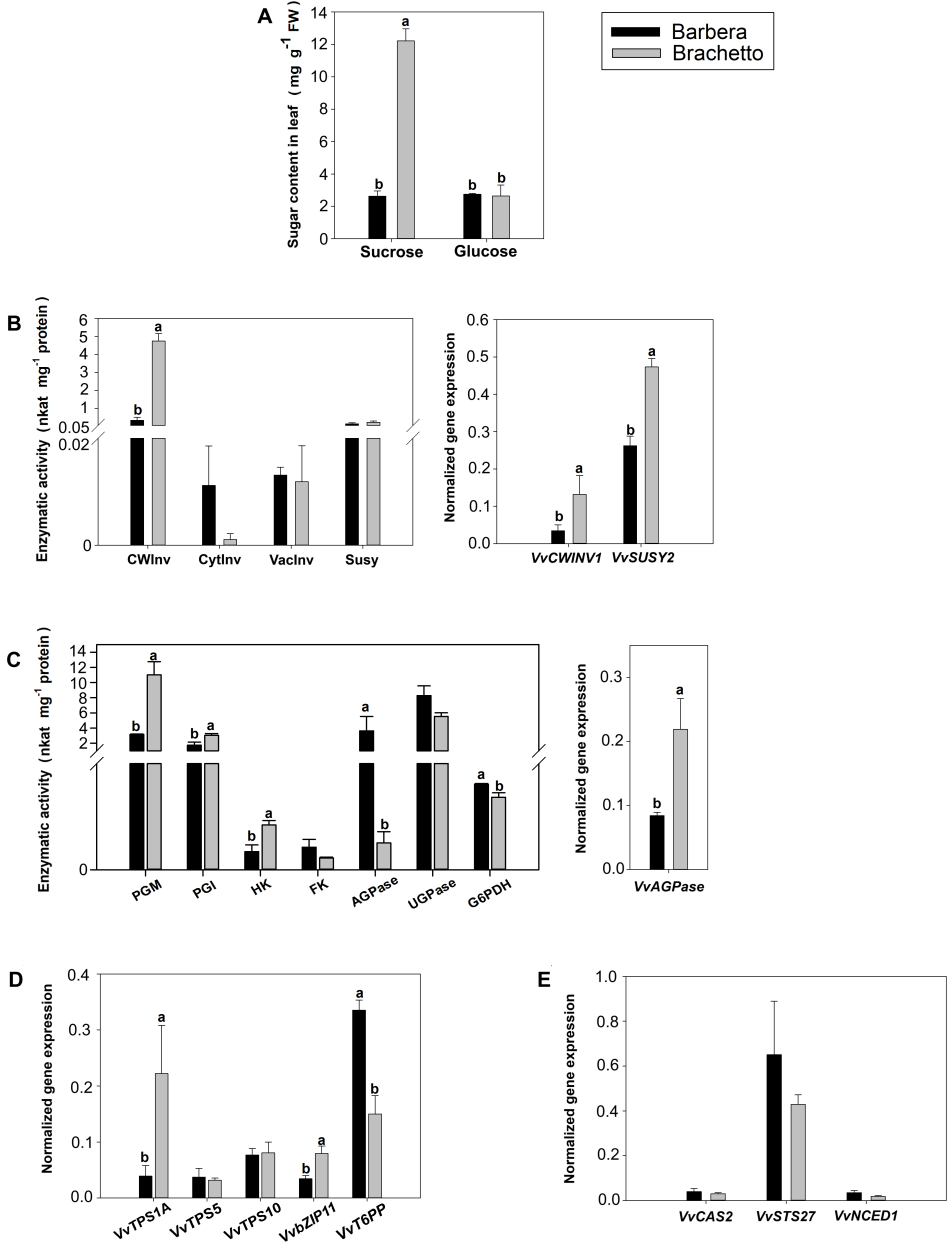
Sugar concentration, enzymatic activities, and gene expression in two grapevine genotypes displaying contrasting susceptibility to Flavescence dorée phytoplasma (FDp), the susceptible ‘Barbera’ and the tolerant ‘Brachetto’. (A) Concentration of soluble sugars. (B, C) Enzymatic activity (left) and gene expression (right) of sucrose (B) and hexose (C) metabolism. (D, E) Expression of genes involved in trehalose-6-phosphate metabolism and signalling (D) and defence from FDp infection (E). Bars are standard errors of the mean (*n*=3) and lower-case letters above bars, when present, represent statistically significant differences between the cultivars at *P*<0.05 as assessed by Student’s *t*-test. AGPase, ADP-glucose pyrophosphorylase; bZIP11, basic leucine zipper transcription factor 11; CAS2, callose synthase; CWInv, cell wall invertase; CytInv, cytosolic invertase; FK, fructokinase; G6PDH, glucose-6-phosphate dehydrogenase; HK, hexokinase; NCED1, 9-*cis*-epoxycarotenoid dioxygenase 1; PGI, phosphoglucoisomerase; PGM, phosphoglucomutase; STS27, stilbene synthase; Susy, sucrose synthase; T6PP, trehalose-6-phosphate phosphatase; TPS, trehalose-6-phosphate synthase; UGPase, UDP-glucose pyrophosphorylase; VacInv, vacuolar invertase.

The sucrose content was significantly higher (up to four times) in the leaves of ‘Brachetto’ with respect to the FDp-susceptible ‘Barbera’. Conversely, glucose concentration did not vary between the two cultivars (Fig. 1A).

The activity signature of key enzymes of carbohydrate metabolism was profiled by a semi-high-throughput method in a microtitre plate format (Jammer *et al.*, 2022). Cell-wall invertase (CWInv) was the main active invertase in grapevine leaves, while cytosolic invertase (CytInv) and vacuolar invertase (VacInv) activity was very low, as previously reported (Cardot *et al.*, 2019). Here, the collected data showed that sucrose and hexose metabolisms were more active in ‘Brachetto’ than ‘Barbera’. Indeed, the activity of CWInv and expression of the corresponding gene and of *VvSusy* (sucrose synthase encoding gene) were significantly higher in the less susceptible genotype, while activity of VacInv and sucrose synthase (Susy) remained unaltered (Fig. 1B). Although a reduced CytInv activity occurred in ‘Brachetto’, this difference was not statically significant. Furthermore, glucose-6-phosphate dehydrogenase (G6PDH) activity was significantly lower in ‘Brachetto’, while activity of hexokinase (HK), phosphoglucoisomerase (PGI) and phosphoglucomutase (PGM) was higher (Fig. 1C). Glucose-1-phosphate utilization to produce uridine diphosphate glucose (UDPG) and adenosine diphosphate glucose (ADPG) was not clearly affected by genotype, as *VvAGPase* transcription rates increased in ‘Brachetto’, while ADP-glucose pyrophosphorylase (AGPase) activity was lower, and no differences were observed for UDP-glucose pyrophosphorylase (UGPase) activity (Fig. 1C).

The expression of *VvTPS1A* (trehalose-6-phosphate synthase biosynthetic gene) and *VvbZIP11* (a sucrose-regulated transcription factor involved in carbohydrate and amino acid metabolism) significantly increased in ‘Brachetto’, accompanied by a steep decrease in *VvT6PP* (trehalose-6-phosphate phosphatase encoding gene) transcription, thereby suggesting a higher concentration of T6P in this cultivar (Fig. 1D). These data indicated that higher sucrose content in ‘Brachetto’ boosted the T6P signalling pathway. As expected for healthy plants, the expression of genes involved in the metabolism of stress-associated hormones [i.e. abscisic acid, *9-cis-epoxycarotenoid dioxygenase 1* (*VvNCED1*)] and in defence responses (stilbene synthase encoding gene *VvSTS27*, callose synthase encoding gene *VvCAS2*) did not change in the two cultivars (Fig. 1E).

### Comparison of Flavescence dorée phytoplasma-infected versus healthy ‘Barbera’ plants in open-field conditions

**Fig. 2.**
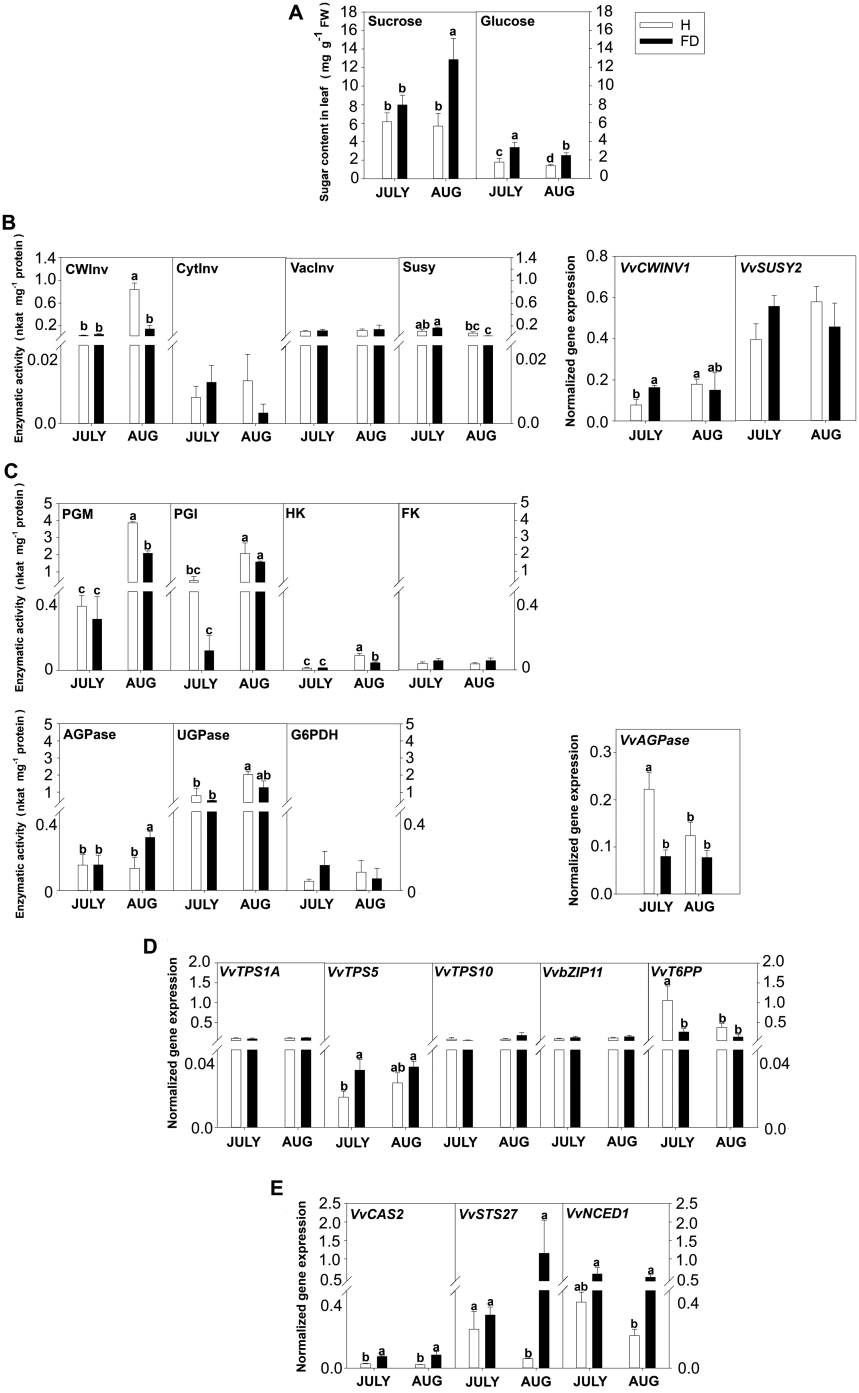
Sugar concentration, enzymatic activities, and gene expression in healthy (H) and Flavescence dorée phytoplasma (FDp)-infected (FD) field-grown ‘Barbera’ plants at two time points during the growing season (15 July and 15 August). (A) Concentration of soluble sugars. (B, C) Enzymatic activity (left) and gene expression (right) of sucrose (B) and hexose (C) metabolism. (D, E) Expression of genes involved in trehalose-6-phosphate metabolism and signalling (D) and defence from FDp infection (E). Bars are standard errors of the mean (*n*=6) and lower-case letters above bars, when present, represent statistically significant differences among conditions and sampling time at *P*<0.05 as assessed by one-way ANOVA (Fisher’s post-hoc test). AGPase, ADP-glucose pyrophosphorylase; bZIP11, basic leucine zipper transcription factor 11; CAS2, callose synthase; CWInv, cell wall invertase; CytInv, cytosolic invertase; FK, fructokinase; G6PDH, glucose-6-phosphate dehydrogenase; HK, hexokinase; NCED1, 9-*cis*-epoxycarotenoid dioxygenase 1; PGI, phosphoglucoisomerase; PGM, phosphoglucomutase; STS27, stilbene synthase; Susy, sucrose synthase; T6PP, trehalose-6-phosphate phosphatase; TPS, trehalose-6-phosphate synthase; UGPase, UDP-glucose pyrophosphorylase; VacInv, vacuolar invertase.

We compared FDp-infected and healthy field-grown plants of the susceptible cv Barbera at an early (15 July) and late (15 August) infection stage, the latter representing the time when the phytoplasma titre typically reaches its peak level (Morone *et al.*, 2007; Roggia *et al.*, 2014). Sucrose concentration in leaf was significantly higher in FDp-infected plants at the late infection stage (15 August), whereas glucose was always more highly accumulated in FDp-infected than healthy plants, regardless of the sampling date (Fig. 2A).

Additionally, analysis of the key enzyme activity showed that sucrose metabolism was not activated by FDp infection. CWInv activity in leaves of healthy plants increased along with time, confirming previous findings (Wu *et al.*, 2015). Activity of CWInv and Susy was similar in healthy and FDp-infected leaves in July, but CWInv activity was significantly lower in FDp-infected samples collected in August (Fig. 2B). While *VvSUSY2* expression reflected activity data, *VvCWINV1* expression was higher in FD-infected plants in July and not in August (Fig. 2B). CytInv and VacInv activity was again very low and showed no significant changes (Fig. 2B).

Hexose metabolism was initiated in the direction of starch synthesis. In fact, HK and PGM activities were lower in FDp-infected plants in August, and no changes were detected for PGI and FK (Fig. 2C). Compared with healthy samples, AGPase activity in FDp-infected plants significantly increased in August, and *VvAGPase* expression was lower in July. Conversely, no significant differences were observed for UGPase and G6PDH activity (Fig. 2C).

The T6P signalling pathway was little affected by FD infection. *VvT6PP* expression was significantly lower in FDp-infected plants in July, whereas *VvTPS5* transcription was induced by the phytoplasmas presence at the same sampling time (Fig. 2D). Expression profiles of *VvTPS1A*, as well as other TPSs such as *VvTPS10*, and of the transcription factor *VvbZIP11* showed no changes (Fig. 2D). As expected, defence responses were activated following pathogen pressure, particularly in August, as underlined by the up-regulation of *VvCAS2*, *VvSTS27*, and *VvNCED1* in FDp-infected samples (Fig. 2E).

Summarizing, we confirmed the increase in sucrose and the activation of defence responses in FDp-infected plants, but sucrose metabolism and TPS expression were not induced.

### Effect of sucrose trunk infusion on Flavescence dorée phytoplasma-infected ‘Barbera’ plants in open-field conditions

**Fig. 3.**
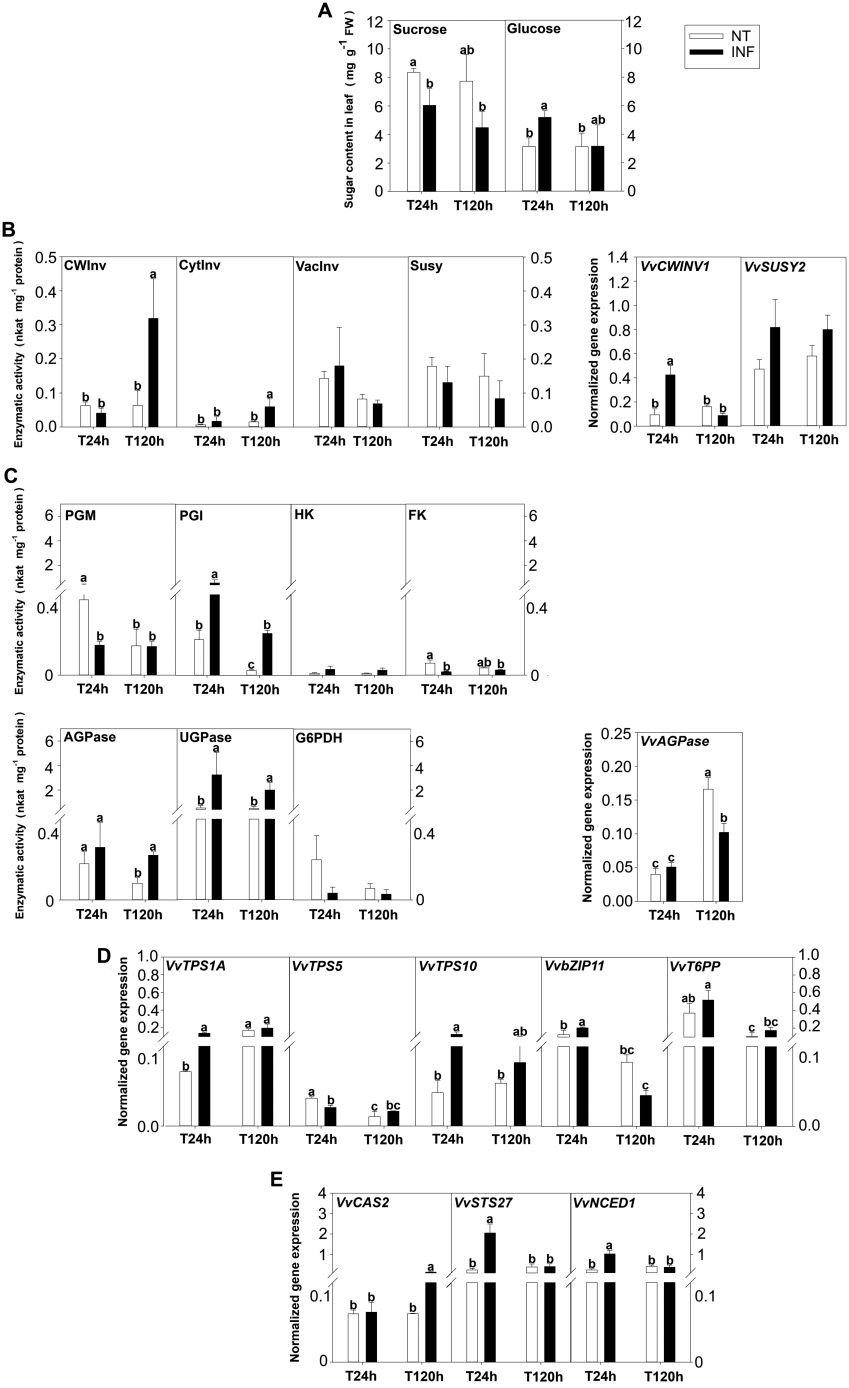
Sugar concentration, enzymatic activities, and gene expression in leaves of Flavescence dorée phytoplasma (FDp)-infected ‘Barbera’ plants, trunk-infused with control buffer (NT) and sucrose solution (INF), assessed 24 h and 120 h after treatment. (A) Concentration of soluble sugars. (B, C) Enzymatic activity (left) and gene expression (right) of sucrose (B) and hexose (C) metabolism. (D, E) Expression of genes involved in trehalose-6-phosphate metabolism and signalling (D) and defence from FDp infection (E). Bars are standard errors of the mean (*n*=5) and lower-case letters above bars, when present, represent statistically significant differences among conditions and sampling time at *P*<0.05 as assessed by one-way ANOVA (Fisher’s post-hoc test). AGPase, ADP-glucose pyrophosphorylase; bZIP11, basic leucine zipper transcription factor 11; CAS2, callose synthase; CWInv, cell wall invertase; CytInv, cytosolic invertase; FK, fructokinase; G6PDH, glucose-6-phosphate dehydrogenase; HK, hexokinase; NCED1, 9-*cis*-epoxycarotenoid dioxygenase 1; PGI, phosphoglucoisomerase; PGM, phosphoglucomutase; STS27, stilbene synthase; Susy, sucrose synthase; T6PP, trehalose-6-phosphate phosphatase; TPS, trehalose-6-phosphate synthase; UGPase, UDP-glucose pyrophosphorylase; VacInv, vacuolar invertase.

To artificially induce an increase in T6P concentration and to boost the related signalling route, we fed grapevine plants of the highly susceptible ‘Barbera’ cultivar with sucrose through xylem infusion. The treatment caused an unexpected decrease in sucrose concentration during the first 24 h after the treatment, which was accompanied by a significant increase in amount of glucose (Fig. 3A). Notably, sucrose infusion induced sucrose degradation as supported by the enhanced CWInv activity at the second sampling time, and by the early up-regulation of *VvCWINV1* (at 24 h), thus anticipating the enzyme activity increase that was observed later (Fig. 3B). Activity of CytInv, VacInv, and Susy remained unchanged, and no difference occurred in *VvSusy* expression (Fig. 3B).

Sucrose infusion activated hexose metabolism. HK and PGI activity increased, while FK and PGI activity were either lower or unaffected in infused plants. Activity of AGPase and UGPase also increased following sucrose infusion, even though *VvAGPase* expression significantly decreased in response to the treatment at the second sampling time (120 h; Fig. 3C).

In parallel, infusion induced the up-regulation of *VvTPS1A*, *VvTPS10*, and *VvbZIP11* 24 h after the treatment, without affecting *VvT6PP* expression (Fig. 3D). Furthermore, it also triggered the plant defence machinery, as indicated by the up-regulation of *VvNCED1* and *VvSTS27* at the first sampling time and of *VvCAS2* at the second one (Fig. 3E). Collectively, these findings highlighted that these metabolic and transcriptional changes, triggered following xylematic sucrose infusion, led to quick sucrose hydrolysis, to activation of defence responses, and potentially of T6P-mediated signalling.

### Effects of mid-shoot girdling on sucrose and trehalose-6-phosphate metabolism and signalling, induction of defence responses, and recovery from Flavescence dorée phytoplasma infection in ‘Barbera’ potted grapevines

Shoot girdling, which temporarily blocks phloem transport, was performed at mid-shoot of pot-grown FDp-infected ‘Barbera’, with the aim of altering sucrose concentration and T6P biosynthesis in either or both of the lower and upper parts of the plant.

**Fig. 4.**
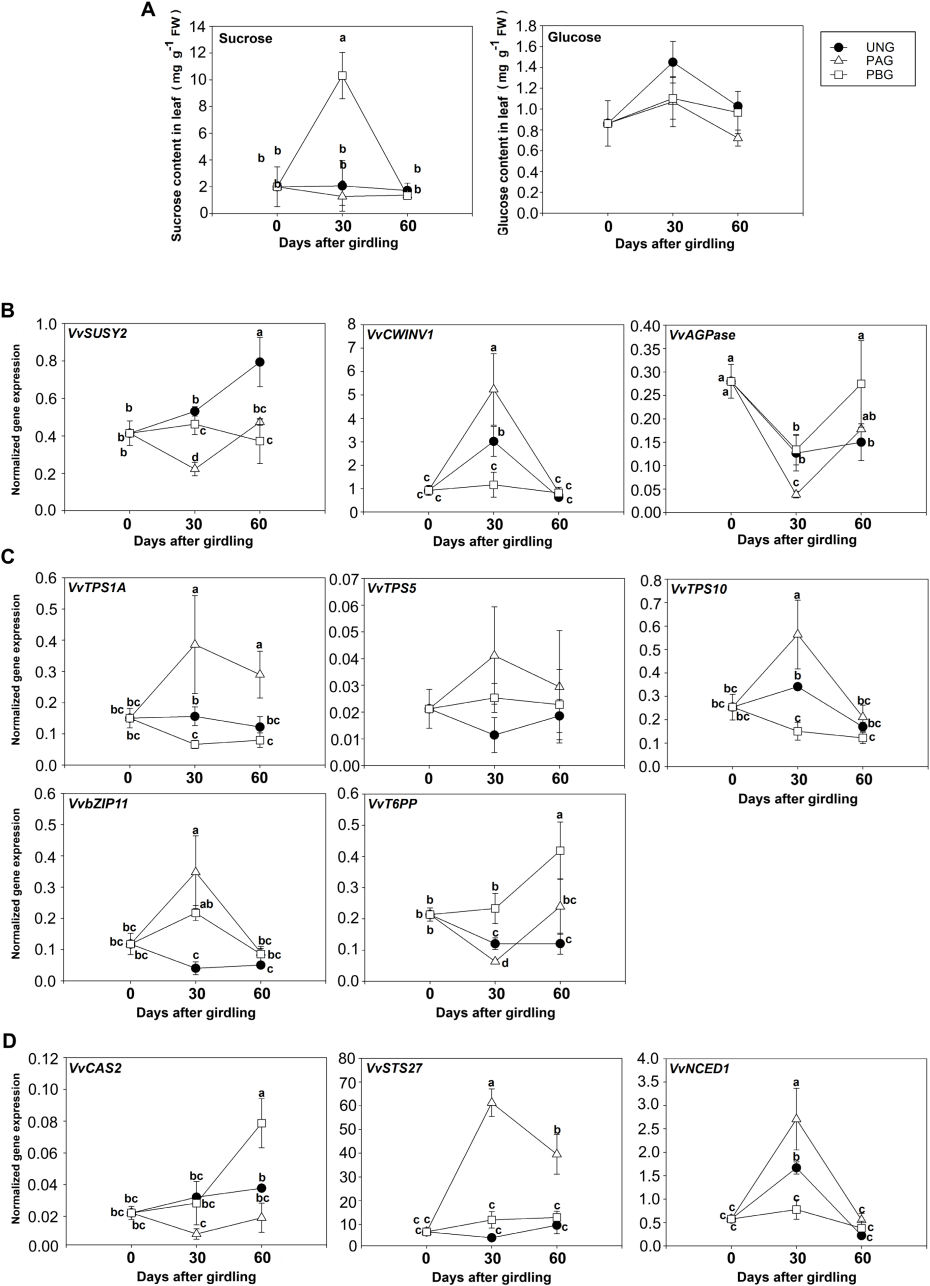
Sugar concentration and gene expression in ungirdled (UNG) ‘Barbera’ plants or in girdled plants, of leaves positioned above the girdle (PAG) or below the girdle (PBG), assessed 30 d and 60 d after the girdling treatment. (A) Concentration of soluble sugars. (B–D) Expression of genes involved in sucrose metabolism (B), trehalose-6-phosphate metabolism (C), and defence against Flavescence dorée phytoplasma infection (D). Bars are standard errors of the mean (*n*=5) and lower-case letters above bars, when present, represent statistically significant differences among conditions and sampling time at *P*<0.05 as assessed by one-way ANOVA (Fisher’s post-hoc test). AGPase, ADP-glucose pyrophosphorylase; bZIP11, basic leucine zipper transcription factor 11; CAS2, callose synthase; CWInv, cell wall invertase; NCED1, 9-*cis*-epoxycarotenoid dioxygenase 1; STS27, stilbene synthase; Susy, sucrose synthase; T6PP, trehalose-6-phosphate phosphatase; TPS, trehalose-6-phosphate synthase.

The girdling treatment was indeed successful in inducing a sucrose increase in leaves positioned below the girdling point (PBG) 30 d after the treatment application, but not later (Fig. 4A). In these leaves, both *VvCWINV1* and *VvSUSY2* were down-regulated, suggesting that sucrose metabolism was not activated (Fig. 4B). *VvAGPase* was also up-regulated (Fig. 4B). Unlike untreated vines, the genes encoding the sucrose biosynthetic enzymes TPS1A and TPS10 were down-regulated following girdling, while expression of the degrading enzyme-encoding gene *VvT6PP* was up to five times higher in the same samples (Fig. 4C). These data suggested that T6P-mediated sucrose signalling was not promoted in PBG leaves. Activation of defence genes was limited to *VvCAS2* but at the last sampling time (60 d post-treatment), as *VvSTS27* did not show significant expression changes over time, while *VvNCED1* was significantly down-regulated at 30 d after girdling (Fig. 4D).

Despite that sucrose and glucose concentrations did not significantly vary in the leaves positioned above the girdle (PAG) (Fig. 4A), *VvCWINV1* was up-regulated in these samples with respect to leaves positioned below the girdle (PBG) and to leaves of the ungirdled plants at 30 d after the treatment (Fig. 4B). Simultaneously, the expression of *VvSUSY2* and *VvAGPase* decreased (Fig. 4B). In PAG leaves, the girdling treatment led to the up-regulation of the *TPS* family genes (*VvTPS1A* and *VvTPS10*), down-regulation of *VvT6PP*, and up-regulation of *VvbZIP11*, pointing to an increase of the T6P signal (Fig. 4C). Excepting *VvCAS2*, both *VvSTS27* and *VvNCED1* were up-regulated in the PAG leaves compared with the ungirdled plants (Fig. 4D). Nonetheless, while *VvSTS27* transcription started to increase at 30 d after girdling and was still significantly higher at the last sampling time, *VvNCED1* transcript amounts were exclusively more abundant at 30 d after the treatment.

**Fig. 5.**
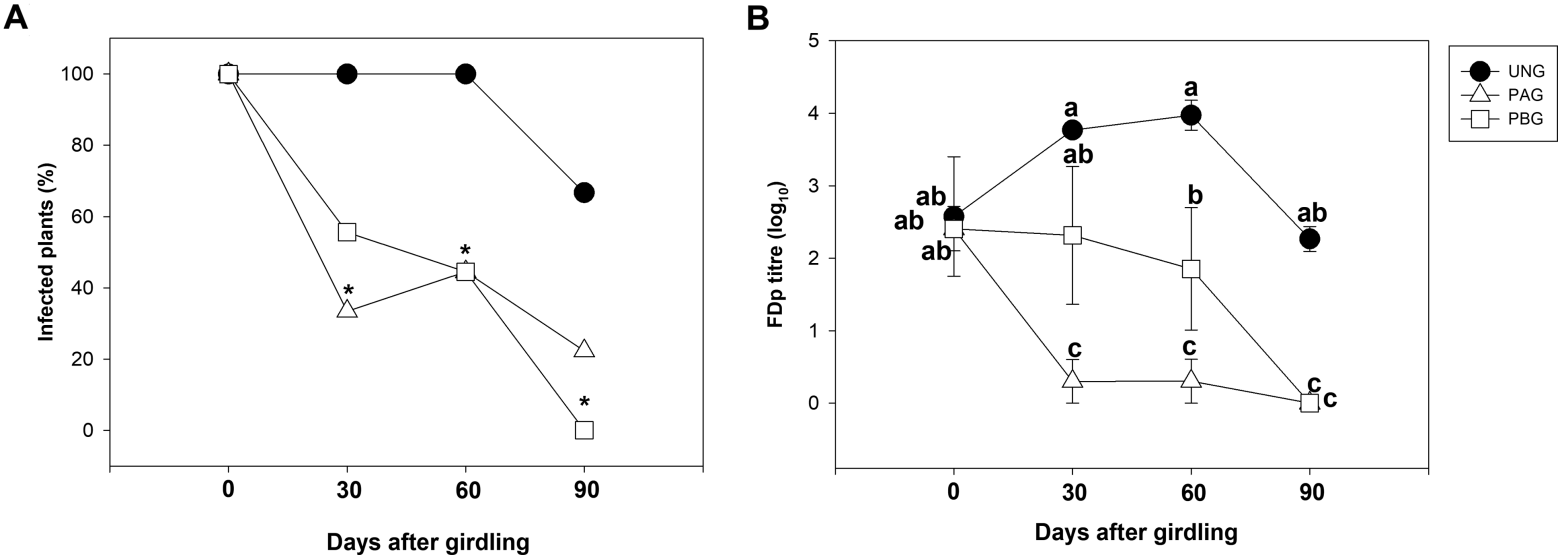
Dynamics of Flavescence dorée phytoplasma (FDp) presence in leaves of FDp-infected ungirdled (UNG) ‘Barbera’ plants or in girdled plants, of leaves positioned above the girdle (PAG) or below the girdle (PBG), assessed 30, 60, and 90 d after the girdling treatment. (A) Percentage of FDp-infected plants. (B) phytoplasma titre. In (A) asterisks represent significant differences (*P*<0.05) from the ungirdled control (UNG) at each time point, as assessed by Z-test. In (B), bars are standard errors (*n*=9) and letters represent significant differences at *P*<0.05 as assessed by one-way ANOVA (Fisher’s post-hoc test).

Since girdling successfully affected TPS expression and sucrose concentration, and activated the plant’s defence responses, we also evaluated its effect on recovery from FD by determining the presence and titre of FDp in leaves. In non-girdled controls, the percentage of plants showing presence of FDp remained stable at 100% for 60 d, then decreased to 64% during the remaining 30 d of experiment (Fig. 5A). On the contrary, following girdling treatment, the percentage of FDp-infected plants started to decrease at 30 d after the treatment, reaching values around 60% and 40% respectively in the PBG and PAG leaves. It then continued to decrease and particularly in the PBG leaves, showed values close to zero at 90 d after the treatment (Fig. 5A). The average phytoplasma load increased in the leaves of non-treated plant up to 60 d after the treatment, following the expected seasonal trend, and then it slightly decreased over time. On the contrary, in both PAG and PBG leaves of girdled plants, the FDp load steadily decreased along the time course, reaching values close to zero at the end of the trial. It should also be mentioned that PAG leaves showed a significantly faster progression of the decrease in FDp load (i.e. reaching the minimum value already at 30 d post-girdling) compared with the PBG leaves (Fig. 5B).

In summary, girdling was most effective in leaves positioned above the girdle, where, even in absence of an increase in sucrose concentration, T6P biosynthesis, defence responses, and recovery were most activated.

## Discussion

### Sucrose and trehalose-6-phosphate metabolisms are differently regulated based on the susceptibility of the cultivar to Flavescence dorée infection

The nature of phloem loading in grapevine source organs is still debated. On one side, expression of *VvSUC27*, which bears sequence similarity with the Arabidopsis sucrose transporter gene *SUC2* whose protein is responsible for phloem uptake, is high in leaves and low in fruits, suggesting an apoplastic mechanism (Afoufa-Bastien *et al.*, 2010). On the other side, diffusion symplastic loading is supported by high expression in mature, but not in young, leaves of the cell wall invertase gene *VvCWINV*, whose protein would metabolize sucrose leaked to the apoplast. Genes for the VvHT1, VvHT3, and VvHT5 plasma membrane hexose transporters, which would allow mesophyll cells to retrieve the resulting hexoses to be channelled to biosynthesis of further symplastic sucrose, are co-expressed with *VvCWINV* (Hayes *et al.*, 2007). Diffusion symplastic loaders display higher leaf concentrations of sucrose than apoplastic loaders in mature leaves, as symplastic sucrose accumulation may help driving the osmotic pressure gradient needed for phloem transport (Fu *et al.*, 2011). Correspondingly, grapevine mature leaves display higher sucrose content than young leaves (Ruffner *et al*., 1990). Therefore, sucrose content and CWInv expression and activity represent a determinant key to the predominant loading pathway in grapevine mature leaves.

**Fig. 6.**
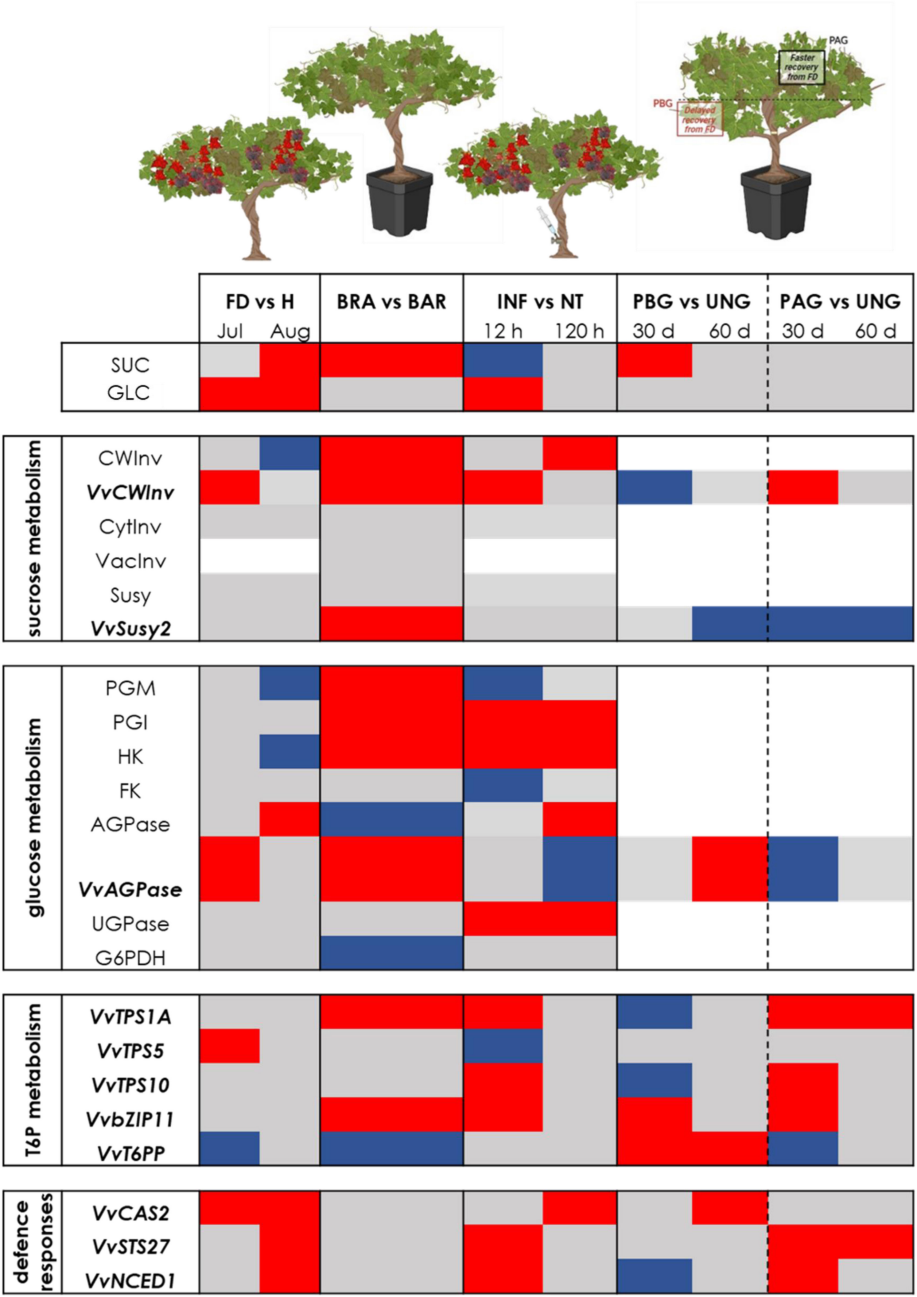
Schematic overview of the main achievements of this study. Heatmap comparing sucrose, hexose, and trehalose-6-phosphate (T6P) metabolism and signalling and defence response gene expression among the different evaluated theses and experiments. The different colours refer to the differential activity and expression of the evaluated enzymes and genes (red indicates activation, blue repression, grey no changes). H, healthy; FD, Flavescence dorée phytoplasma infected; BRA, ‘Brachetto’; BAR, ‘Barbera’; INF, infused with sucrose solution; NT, infused with control buffer; PBG, positioned below the girdling point; PAG, positioned above the girdling point; UNG, non-girdled; AGPase, ADP-glucose pyrophosphorylase; bZIP11, basic leucine zipper transcription factor 11; CAS2, callose synthase; CWInv, cell wall invertase; CytInv, cytosolic invertase; FK, fructokinase; G6PDH, glucose-6-phosphate dehydrogenase; HK, hexokinase; NCED1, 9-*cis*-epoxycarotenoid dioxygenase 1; PGI, phosphoglucoisomerase; PGM, phosphoglucomutase; STS27, stilbene synthase; Susy, sucrose synthase; T6PP, trehalose-6-phosphate phosphatase; TPS, trehalose-6-phosphate synthase; UGPase, UDP-glucose pyrophosphorylase; VacInv, vacuolar invertase.

In a previous work, we screened different grapevine genotypes for susceptibility to FDp infection, showing that ‘Brachetto’ is less susceptible than ‘Barbera’ (Ripamonti *et al.*, 2021). Here, we characterized sugar concentration, and the activity and expression of sucrose- and hexose-metabolizing enzymes in mature leaves of healthy plants of these genotypes (Fig. 1). Our results show that sucrose concentration is markedly higher in ‘Brachetto’, and these changes are paralleled by patterns of CWInv activity, suggesting that the incidence of symplastic versus apoplastic loading may be genotype-specific in grapevine (i.e. higher in ‘Brachetto’ than in ‘Barbera’). In the ‘Brachetto’ variety, which showed very high CWInv activity, also the activity of hexose phosphorylating enzymes was enhanced (Fig. 6). These results suggest that hexoses produced by cleavage of apoplastic sucrose would be phosphorylated in the mesophyll cell and channelled to the production of sugar nucleotides, and to the biosynthesis of T6P. Up-regulation of *TPS* genes was indeed observed in ‘Brachetto’, confirming the connection with sucrose concentration observed in many plants (Zhang *et al.*, 2009; Figueroa and Lunn, 2016; Morabito *et al.*, 2021).

### Flavescence dorée phytoplasma infection increases sucrose concentration but inhibits sucrose metabolism

In leaves of FDp-infected ‘Barbera’ plants, sucrose and glucose concentration were higher than in leaves of healthy plants, confirming previous reports on phytoplasma-infected plants, including grapevine (Lepka *et al.*, 1999; Prezelj *et al.*, 2016). The increase in sugar concentration occurring in FD versus H plants was more evident in August, when phytoplasma load normally reaches its maximum (Prezelj *et al.*, 2013; Roggia *et al.*, 2014). T6P biosynthesis was not affected but expression of the T6P degradation gene *VvT6PP* decreased, and this may suggest that the T6P signal was up-regulated as expected in the presence of higher sucrose concentration (Figueroa and Lunn, 2016) (Fig. 6).

Sucrose accumulation in phytoplasma-infected leaves could be due to different reasons. Callose deposition at sieve plates may slow down phloematic transport (Santi *et al.*, 2012), thus containing phytoplasma spread, but also hindering phloematic sugar export from source leaves (Musetti *et al.*, 2013). Sucrose accumulation can also be induced by variation in the expression and activity of sucrose-cleaving enzymes, which is commonly modulated under pathogen infection (Proels and Hückelhoven, 2014), including in grapevine (Hayes *et al.*, 2010).

As previously shown for other grapevine genotypes, in ‘Barbera’ leaves the main sucrolytic activity is contributed by CWInv, followed by Susy (Wu *et al.*, 2015; Prezelj *et al.*, 2016; Cardot *et al.*, 2019), while activities of other invertases were low and not affected by the infection development. Activity of CWInv and expression of *VvCWINV* were higher in FD plants in July (Fig. 6). In a similar experimental set-up, Prezelj *et al.* (2016) reported a slight, though not significant, decrease of CWInv activity. Activation of apoplastic sucrolytic enzyme activity is often observed in leaf tissues challenged with pathogens, and, if coupled to uptake of the resulting hexoses in the cytoplasm, may allow metabolic provision for cells actively engaged in defence responses, and hexose starving of apoplastic pathogens (Roitsch *et al.*, 2003; Berger *et al.*, 2004; Naseem *et al.*, 2017). However, in interactions with strictly symplastic pathogens such as phytoplasmas, this would represent a shortcoming for the plant, as carbon availability for the pathogen would increase (Liu *et al.*, 2022). In conformity, we observed that at the August sampling date, when the phytoplasma load is normally the highest (Prezelj *et al.*, 2013; Roggia *et al.*, 2014), activity of apoplastic invertase was lower in FDp-infected plants. Accordingly, at this stage also glucose phosphorylation and hexose-P interconversion activity were lower in infected plants (Fig. 2). The modulation of CWInv expression thus appears to follow a biphasic pattern: at an earlier stage of infection (our first sampling date), CWInv activation could provide hexoses for plant metabolism and callose plug deposition, as proposed by Santi *et al.* (2012) in *Bois noir*-infected vines. At a more severe stage of the disease (i.e. August), since hexose phosphates are required by phytoplasmas for their metabolism (André *et al.*, 2005), plant responses would shift from physically hindering phytoplasma spread to active repression of the sucrose-cleaving enzyme CWInv and of hexose phosphorylating enzymes. This strategy could thereby allow the plant to contain the phytoplasma proliferation by reducing cellular hexose-P availability. At this stage FD also triggers AGPase activity, possibly inducing starch synthesis, and thus causing further diversion of the hexose-P metabolic flow. A similar observation was also made by Prezelj *et al.* (2016), though differences between healthy and FDp-infected vines were not significant in that study. Repression of sucrolytic enzyme activity would also support phloem loading by increasing the source leaf apoplastic sucrose concentration.

Expression and activity of CWInv are induced by soluble sugars (Roitsch *et al.*, 2003). Therefore, it is conceivable that during the first stage of infection (July) elevated sucrose is induced by mechanical hindrance of phloem transport, and the increase in hexoses produced by constitutive sucrolytic enzymes may in turn up-regulate *CWInv*-encoding transcripts. Alternatively, pathogens have been demonstrated to act by the production of effectors harnessing plant cell mechanisms and affecting sugar availability (Naseem *et al.*, 2017). Phytoplasmas can produce effectors with demonstrated action on plant proteins (Sugio *et al.*, 2011; Kirazawa *et al.*, 2022). Both these models would, however, not mechanistically accommodate the inhibition of CWInv observed at the late stage of infection. This effect may be due to unknown plant defence signals acting on sucrolytic enzyme-encoding genes and occurring at the transcriptional and/or post-transcriptional level or interacting antagonistically with putative phytoplasma effectors.

It also emerged that the phytoplasma presence triggered the expression of defence response genes. Nevertheless, T6P metabolism and signalling were down-regulated under infection (Fig. 2). We hypothesized that, even though some key defence genes were up-regulated, repression of the sucrose- and T6P-related signalling cascade might promote maintenance of infection status at the expense of recovery.

### The trehalose-6-phosphate signalling route is associated with expression of key defence genes and with reduced Flavescence dorée phytoplasma load in leaves of infected grapevines

No complete resistance towards FDp has been detected in *V. vinifera*; however, the observation that different genetic levels of FDp susceptibility exist (Eveillard *et al.*, 2016; Ripamonti *et al.*, 2021) and that diseased plants can recover (Roggia *et al.*, 2014) implies that grapevine can mount defence responses against the pathogen. Known molecular mechanisms of recovery from FD and other grapevine phytoplasmas include activation of callose synthase genes, which allows formation of callose plugs to limit phloematic spread of the phytoplasma (Santi *et al.*, 2012), modulation of oxidative stress and of the ABA and ethylene signals (Gambino *et al.*, 2013), and accumulation of secondary metabolites such as anthocyanins (Margaria *et al.*, 2014), terpenoids (Teixeira *et al.*, 2020), and stilbenes (Pagliarani *et al.*, 2020). Stilbenes are grapevine-specific phenolic compounds, highly accumulated in response to abiotic and biotic stresses (Vannozzi *et al.*, 2012). The *VvCAS2*, *VvNCED1*, and *VvSTS27* genes, which respectively control callose, ABA, and stilbene biosynthesis, are overexpressed in plants recovering from FDp infection (Pagliarani *et al.*, 2020).

The observation that healthy plants of ‘Brachetto’ have 4-fold higher sucrose concentration and more sustained T6P biosynthesis than ‘Barbera’ (Fig. 1), where defence responses were activated, and that sucrose concentration increases and T6P degradation decreases in FD-infected ‘Barbera’ plants suggests that sucrose could contribute to control FDp load by activating the T6P signalling pathway (Fig. 2). We tested this hypothesis by inducing T6P biosynthesis with treatments designed to increase sucrose concentration in diseased plants, using either xylem sucrose infusion or a girdling treatment. We were not able to measure any increase in sucrose concentration in the leaves in response to xylem sucrose infusion, probably due the fact that a transient increase, undetected in our sampling scheme, led to the activation of CWInv activity (Roitsch *et al.*, 2003). However, following the treatment, we observed activation of T6P biosynthesis coupled to up-regulation of defence-associated genes (Figs 3, 4, 6). Also, in the case of girdling, a dynamic interaction between sucrose levels and regulation of sucrolytic enzymes seemed to be in action. In leaves positioned above the girdling point (PAG) we observed no increase in sucrose but activation of *VvCWINV* expression, while the opposite occurred in leaves positioned below the girdling (PBG). This result suggests that an early and transient increase in sucrose (though undetected in our experimental condition) would favour the activation of sucrolytic activity and the decrease of sugar levels in PAG leaves, whereas a transient sucrose decrease in PBG leaves would induce the opposite response. Most importantly, however, girdling clearly triggered the T6P signal in PAG leaves, consistently with the hypothesis of an initial and transient increase in sucrose (Fig. 4). These data also correlated with the sustained expression of defence-related genes and with faster recovery dynamics based on the quantification of the FDp titre (Fig. 5).

Based on these results, we conclude that the sucrose-activated T6P signal may impact FDp multiplication and survival. We acknowledge that transcript abundance is not necessarily translated into changes in enzyme activity, and that correlations between sucrose and T6P can be strongly influenced by underlying shifts in metabolic status as well as developmental changes and responses to environmental conditions. However, several lines of evidence from this study support this conclusion: (i) T6P biosynthesis is more active in the less susceptible genotypes, (ii) FDp infection slows down T6P degradation and activation of defence genes; and (iii) treatments that increase T6P biosynthesis cause activation of defence responses and, in the case of girdling, of recovery from FD. There thus emerges a primary role of the T6P signal as a constitutive mark in tolerant plants and as an infection-induced signal that may function to control the phytoplasma load.

In source leaves, T6P activates phosphoenolpyruvate carboxylase and nitrate reductase, hence diverting carbon to biosynthesis of organic acids and amino acids. The increase in T6P levels in FDp-infected grapevine leaves could thus contribute to the scavenging of hexose phosphates required for phytoplasma metabolism and multiplication, representing an efficient countermeasure to phytoplasma virulence. In addition, since T6P seems to contribute to elicitation of molecular defence responses, systemic signalling to transcription factors is envisaged. This is supported by our finding that, while girdling enhanced both T6P biosynthesis and defence responses, leading to a faster recovery of the leaves above the girdling point, in the leaves below the treatment application the T6P signalling route was turned off. This condition was, however, associated with the triggering of some defence responses at the molecular level (i.e. increased expression of *VvCAS2*) and of recovery, although this occurred at a slower pace than in leaves above the girdle. These findings supported the establishment of a biochemical signal inducing recovery, which initiated above the girdling point, then apparently spread to leaves located below the girdling. Girdled woody plants reconstitute bark tissues and phloematic connections after a few weeks (Chen *et al.*, 2014), implying that such a message may be delivered through the phloem vasculature to reach leaves below the girdling once the bark is reconstituted. This could explain the delay in recovery we observed in leaves positioned below the girdling. Additionally, since *VvNCED1* was up-regulated in the leaves above the girdling, and ABA does move from source leaves within the phloem (McAdam *et al.*, 2016), it could represent a candidate for delivering such a message. This subject however needs further deepening, as many other substances, including proteins, peptides, mRNAs, and miRNAs, are phloem-mobile, and could convey the signal induced by T6P.

## Supplementary data

The following supplementary data are available at *JXB* online.

Fig. S1. The infusion treatment in progress.

Fig. S2. Detail of the girdled shoots 10 d after the girdling treatment.

Table S1. Primers used in the study.

Table S2. Two-way ANOVA results (*P*-values).

erae381_suppl_Supplementary_Material

## Data Availability

The data that support the findings of this study are available in the supplementary data of this article.
