# Supplementary material for: The sucrose signalling route controls Flavescence dorée phytoplasma load in grapevine leaves
Source: J Exp Bot. 2024 Sep 11;76(11):3071–87. doi: 10.1093/jxb/erae381 (PMC12321740; doi:10.1093/jxb/erae381)
Supplement: erae381_suppl_Supplementary_Material [file erae381_suppl_supplementary_material.pdf]

The sucrose signalling route controls *Flavescence dorée* phytoplasma load in grapevine leaves

Cristina Morabito[1*,] Chiara Pagliarani[2], Claudio Lovisolo[1], Matteo Ripamonti[1,2,a], Domenico Bosco[1], Cristina Marzachì[2], Thomas Roitsch[3,4], Andrea Schubert[1]

[1]PlantStressLab, University of Turin, Department of Agricultural, Forestry, and Food Sciences, Grugliasco, Italy; [2]Institute for Sustainable Plant Protection, CNR, Turin, Italy; [3]Dept of Plant and Environmental Sciences, University of Copenhagen, Denmark; [4]Global Change Research Institute of the Czech Academy of Sciences, Brno, Czech Republic

[a] Present address: Environmental Research and Innovation Department (ERIN), Luxembourg Institute of Science and Technology (LIST), Esch-sur-Alzette, Luxembourg.

*Corresponding author

Cristina Morabito: cristina.morabito@unito.it

Chiara Pagliarani: chiara.pagliarani@ipsp.cnr.it

Claudio Lovisolo: claudio.lovisolo@unito.it

Matteo Ripamonti: matteo.ripamonti@list.lu

Domenico Bosco: domenico.bosco@unito.it

Cristina Marzachì: cristina.marzachi@ipsp.cnr.it

Thomas Roitsch: roitsch@plen.ku.dk

Andrea Schubert: andrea.schubert@unito.it

Running title: **Sucrose role in FDp-grapevine interaction.**

Supplementary data: 2 tables, 2 figures.

Table S1 – Primers used in this study

Gene name	Gene ID (CRIBI)	Primer name	Forward primer sequence	Reverse primer sequence	Amplicon size (bp)
VvTPS1_chr10 (Morabito *et al*., 2021)	VIT_10s0003g02160	***VvTPS1_A***	CTCACAAGCCAGGTTCATGA	GGCCACAAACTCATAGCTAACA	192
VvTPS5 (Morabito *et al*., 2021)	VIT_02s0012g01680	***VvTPS5***	GTGTTCTTGCCAATGAGCCA	TGCCGCATTGTTACAAGGAG	115
VvTPS10 (Morabito *et al*., 2021)	VIT_01s0026g00280	***VvTPS10***	TTATGCTCGCCACTTCCTGT	CACAGTCCGACCCGAGTAAT	100
VvT6PP (Morabito *et al*., 2021)	VIT_16s0022g00660	***VvT6PP***	GCTGGTAGTCATGGGATGGA	AAAATTCACTCGCAGGCTGG	130
VvbZIP11 (Morabito *et al*., 2021)	VIT_18s0001g13040	***VvbZIP11***	CCTCAATTACATGAACACAAGCA	TGATGATGACACGAAGGAGGA	160
VvCWINV1 (Hayes *et al*., 2007)	VIT_09s0002g02320	***VvCWINV1***	TCTATCAACAGCTCTACGGGT	TCTCACGGTTGTAGCTTCCA	124
VvSUSY2 (Morabito *et al*., 2021)	VIT_07s0005g00750	***VvSUSY2***	GCCCTGCATGGTTCAATTGA	GTCAAGCCTTGCCATGGAAA	114
VvCAS2 (Santi et al. 2012)	VIT_06s0004g01270	***VvCAS2***	TTCACCCCAGTTGCATTTCT	CCGATCCTTCCTATGACCAC	129
VvNCED1 (Ferrero et al. 2018)	VIT_19s0093g00550	***VvNCED1***	GGTGGTGAGCCTCTGTTCCT	CTGTAAATTCGTGGCGTTCACT	132
VvSTS27 (Vannozzi et al., 2012)	VIT_16s0100g00990	***VvSTS27***	CAGGTGGAACTGTCCTTCGAA	ACAAGAACTCGTGCTCCTGCAT	67
VvActin (Gambino *et al*., 2011)	VIT_04s0044g00580	***VvACT***	GCCCCTCGTCTGTGACAATG	CCTTGGCCGACCCACAATA	101
VvUbiquitin (Morabito *et al*., 2021)	VIT_16s0098g01190	***VvUBI***	TGAGGCTTCGTGGTGGTATT	GCGGCAGATCATTTTGTCCT	80

Gambino G Minuto M Boccacci P Perrone I Vallania R Gribaudo I (2011) - Characterization of expression dynamics of WOX homeodomain transcription factors during somatic embryogenesis in Vitis vinifera. *Journal of Experimental Botany* 62 1089–1101

Ferrero M, Pagliarani C, Novák O, Ferrandino A, Cardinale F, Visentin I, Schubert A (2018) - Exogenous strigolactone interacts with abscisic acid-mediated accumulation of anthocyanins in grapevine berries. *Journal of Experimental Botany* 69, Issue 9, 2391–2401

Table S2 – P-values obtained from the two-way ANOVA analyses, grouped by the different experiments presented in the work (reference to the corresponding figure in the column title). In bold the measurements showing significant interaction among the considered variables.

**Data Fig. 2**		**Data Fig. 3**		**Data Fig. 4**		**Data Fig. 5**	
	**P-value**		**P-value**		**P-value**		**P-value**
Sucrose	0.074	Sucrose	0.747	**Sucrose**	**<0.001**	FDp load	0.116
Glucose	0.399	Glucose	0.326	Glucose	0.07		
**CW Inv**	**0.006**	CW Inv	0.056	***VvCWINV1***	0.031		
Cyt Inv	0.243	Cyt Inv	0.438	*VvSUSY2*	0.587		
Vac Inv	0.914	Vac Inv	0.795	*VvAGPase*	0.504		
**Susy**	**0.037**	Susy	0.861				
VvCWINV1	0.124	***VvCWINV1***	**0.012**	*VvTPS1A*	0.571		
VvSUSY2	0.17	*VvSUSY2*	0.465	*VvTPS5*	0.577		
				VvTPS10	0.056		
**PGM**	**<0.001**	PGM	0.14	***VvbZIP11***	0.276		
PGI	0.891	PGI	0.054	*VvT6PP*	0.101		
**HK**	**0.034**	HK	0.874				
FK	0.892	FK	0.118	***VvCAS2***	**0.049**		
				VvSTS27	**<0.001**		
AGPase	0.177	AGPase	0.722	***VvNCED1***	**0.03**		
UGPase	0.558	UGPase	0.568				
G6PDH	0.351	G6PDH	0.345				
VvAGPase	0.264	***VvAGPase***	**0.017**				
VvTPS1A	0.432	*VvTPS1A*	0.571				
VvTPS5	0.565	*VvTPS5*	0.067				
VvTPS10	0.556	*VvTPS10*	0.304				
VvbZIP11	0.827	***VvbZIP11***	**0.007**				
VvT6PP	0.581	*VvT6PP*	0.678				
VvCAS2	0.377	***VvCAS2***	**0.035**				
VvSTS27	0.109	*VvSTS27*	0.075				
VvNCED1	0.512	*VvNCED1*	0.339				

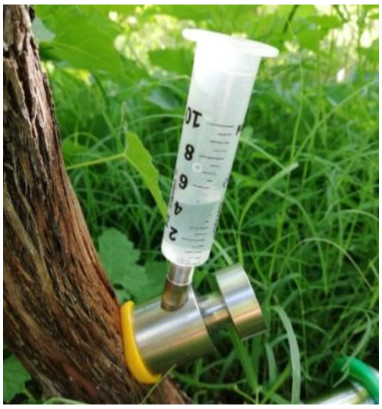

Fig. S1 –The infusion treatment in progress.

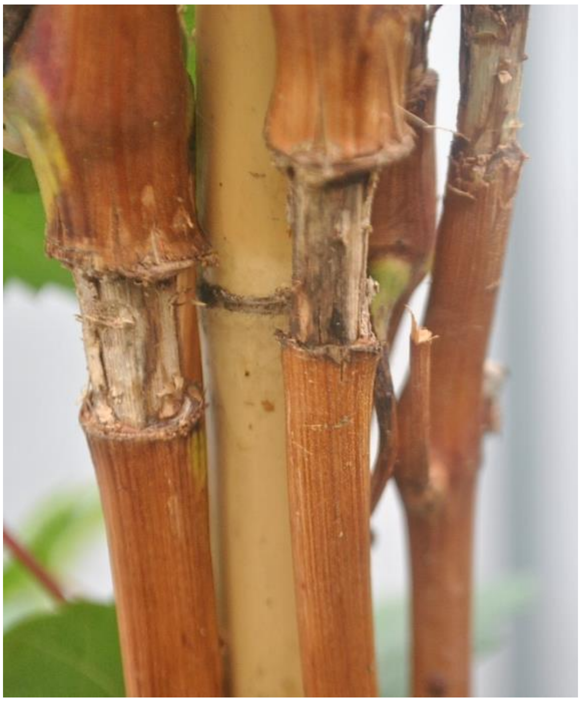

Fig. S2 – Detail of the girdled shoots 10 days after the girdling treatment.
